# Change in screen time and overuse, and their association with psychological well-being among US-wide school-age children during the COVID-19 pandemic: analysis of the National Survey of Children’s Health (NSCH) years 2018–21

**DOI:** 10.1186/s13034-023-00688-7

**Published:** 2024-01-13

**Authors:** Helena T. Wu, Jiandong Li, Amy Tsurumi

**Affiliations:** 1 University of Chicago, Chicago, IL USA; 2 Department of Surgery, Massachusetts General Hospital, 50 Blossom St., Their 340, Boston, MA 02114 USA; 3 Central University of Finance and Economics, Beijing, China; 4 Harvard Medical School, Boston, MA USA; 5 Shriners Hospitals for Children-Boston, Boston, MA USA

**Keywords:** Screen time, Screen addiction, Screen overuse, COVID-19, Pandemic, Psychological well-being, Mental Health and Wellbeing, Child health, Adolescent health, Emerging infectious diseases, Epidemiology, Infectious disease

## Abstract

**Background:**

Previous studies on screen use and children’s mental health during the Coronavirus Disease 2019 (COVID-19) pandemic focused only on the timeframe during the pandemic, on children between narrow age ranges, only among a subset of children who have previously reported COVID-related severe family economic hardship or worries, or did not distinguish between instructive versus recreational device usage. Thus, in this study, we analyzed trends, specifically related to recreational screen use, and associations with psychological well-being (PWB) in the years before versus during the COVID-19 pandemic, among a wide range of school-aged children, widely across the nation.

**Methods:**

Using the National Survey of Children’s Health (NSCH) years 2018–21, we analyzed a large random sample of school-aged children (6–17 years old) across the US (n = 88,823). We developed PWB issue scores (PWBIS) using self-reported measures relevant to this age group, and constructed regression models to assess the magnitude of the contribution of the pandemic on recreational screen use and PWB.

**Results:**

The prevalence of recreational screen overuse and PWBIS increased significantly during the pandemic, compared to prior years. We also detected a notable effect of the pandemic on increased PWBIS, as well as its interaction term finding that it strengthened the association between screen time and PWBIS (p < 0.01 across all regression models).

**Conclusions:**

Accordingly, our results demonstrate the importance of the pandemic itself as an independent adverse factor and effect measure modifier for screen overuse and PWB more generally among all school-age children widely across the US. Our study used the most current data available, and future studies to evaluate whether these effects are persistent in the years after the pandemic are important.

**Supplementary Information:**

The online version contains supplementary material available at 10.1186/s13034-023-00688-7.

## Background

Studies on children and adolescents’ psychological well-being (PWB) have generally found a negative association with increased screen time [1–10]. Overall screen use from video and online games, television, internet, smartphones, and online education increased, especially during the coronavirus disease of 2019 (COVID-19) pandemic year of 2020 [11, 12]. Previous studies have investigated the effect of the pandemic on screen time and its associations with mental health among children [13, 14]. However, these studies evaluated associations only during the pandemic, and among a subset of children previously reporting severe family and economic impact of COVID-19 [13] or “COVID-related worry,” [14] within specific age ranges. Thus, a study including all school-age children (6–17 years old) widely across the United States (US) representing all demographics and economic status, and analyzing the relationship between screen time and PWB over time, before and during the pandemic, is important.

Since the pandemic generally increased screen time among school-age children due to online courses, we aimed to investigate recreational screen time (*i.e.* outside of school-related instructive screen use) more specifically, as we postulated that voluntary excessive screen overuse may be especially alarming during the time when school was mostly conducted online. Use of smartphones and other electronic devices has been shown to have both positive and negative effects [1, 15]. There is no definitive cut-off to determine hours of excessive screen use, and the specific type or content could have different impact. However, the American Academy of Pediatrics (AAP)’s current guideline for children aged 2–5 years old is to limit non-educational screen time to 1 h per day, and for children aged 6 years and older, to ensure that screen time does not interfere with sleep, physical activity, and other healthy behaviors [16, 17].

The American Psychiatric Association (APA) classifies internet gaming addiction as an impulse disorder [18], and as of 2018, the World Health Organization (WHO) included gaming disorder within the International Classification of Diseases, 11th revision (ICD-11) [19]. On the other hand, screen addiction from smartphones and other electronic devices has not yet been classified as a clinical disorder, even though the harms of screen overuse specifically related to these devices are well documented [20, 21]. Assessments of behavioral addiction include functional impairment or distress and persistence of the behavior over time [22]. Various addiction screening tools specifically for smartphone or internet addiction have been proposed and used in research studies [4, 20], and previous studies have reported associations between increased screen time and indicators of addiction [9, 23–25]. Thus, further studies addressing the negative impact of screen time and overuse on PWB is important.

The objective of this study is to explore the impact of the pandemic on recreational screen time and overuse, and its association with various measures of children’s PWB, using the National Survey of Children’s Health (NSCH), a large sample of children widely across the US. This survey includes various measures most relevant to this age group that could be used to assess PWB, highlighting the advantage of our study design aimed at understanding children’s conditions. We explored whether the trends and association between screen overuse and PWB have evolved during the year before versus during the pandemic, as well as the impact of the pandemic as an effect measure modifier to strengthen the association.

## Methods

### Study subjects

**Fig. 1:**
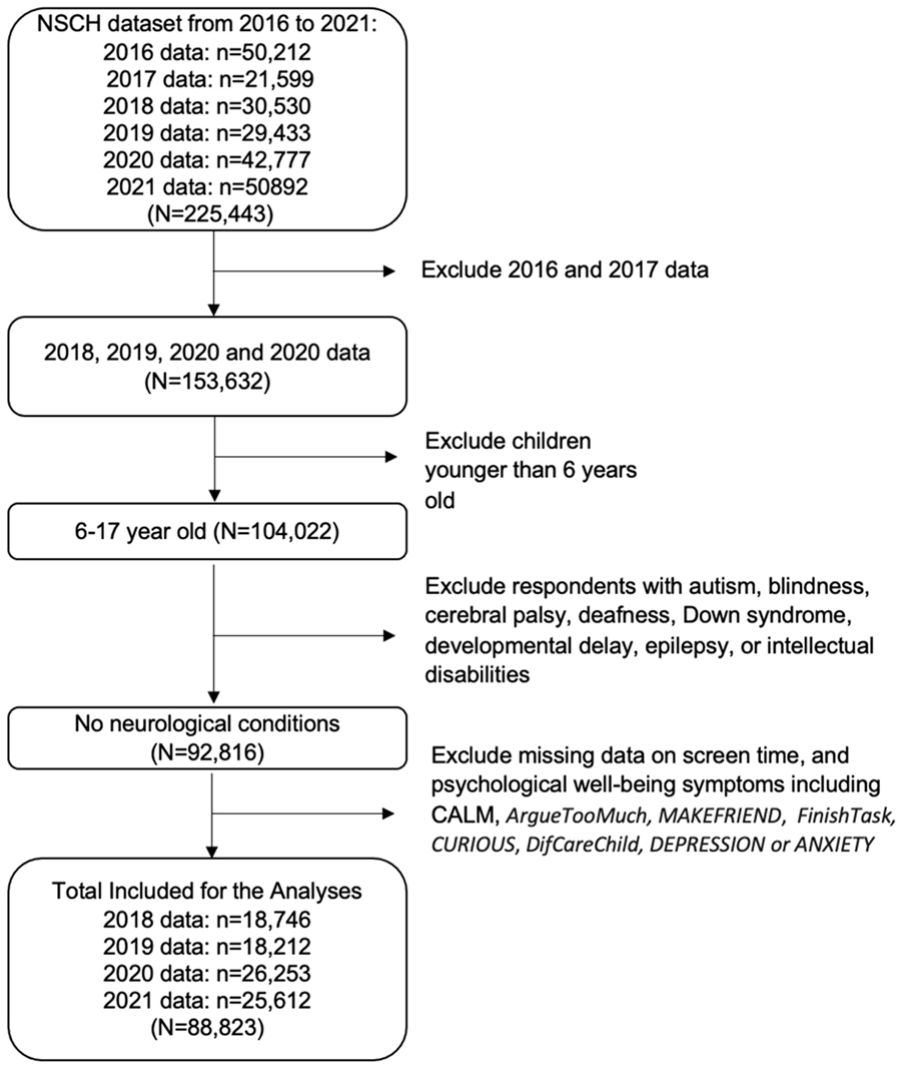
Flowchart of study inclusion/exclusion criteria

Since 2016, the Census Bureau has been administrating the National Survey of Children’s Health (NSCH) by mail and web-based surveys (instead of the earlier telephone method) in 50 states and the District of Columbia. Questionnaires are sentto one randomly chosen child aged 0–17 years, or their caregiver, per household. The publicly available dataset includes 50,212 observations for 2016, 21,599 for 2017, 30,530 for 2018, 29,433 for 2019, 42,777 for 2020, and 50,892 for 2021 [26]. Since our objective is to investigate school-age children, we included those aged 6–17 years widely. For years 2016 and 2017, the NSCH collected data on computer and TV hours separately, and total screen time can be assessed by adding those hours together. From 2018, the NSCH combined the survey question on computer and TV hours to collect screen time as one measure. We found that screen time range for 2016 and 2017 to be 0– ≥ 8 h, and 2018–2021 to be 0–4 h, providing the evidence that the data is not comparable. Therefore, we included only data from 2018, 2019, 2020, and 2021 to maintain consistency in the methods to collect data on screen time. We excluded respondents with reported autism, blindness, cerebral palsy, deafness, Down syndrome, developmental delay, epilepsy, or intellectual disabilities, as PWB measures and screen time behavior may require different assessments. Furthermore, we excluded missing data on screentime or PWB symptoms. In summary, we analyzed a total of 88,823 observations (18,746 respondents for 2018, 18,212 for 2019, 26,253 for 2020, and 25,612 for 2021) (Fig. 1).

### Variables and measures used

#### Description of NSCH data

Details on the NSCH study variable names according to the code book, questions used to collect data, and descriptions of recoded variables used in our study for subsequent analyses are provided in Additional file 1: Table S1. The 4 year weight for 2018–2021 was obtained from the NSCH’s Guide to Multi-Year Analysis, and adjusted weight was used for the analyses.

#### Subject demographics

We obtained data on age, race, ethnicity, sex, and poverty ratio. In accordance with the NSCH’s Guide to Analysis with Multiply Imputed Data, we computed the poverty ratio by averaging across the six imputed poverty ratio values, for subsequent analyses.

#### Recreational screen time measure

**Fig. 2:**
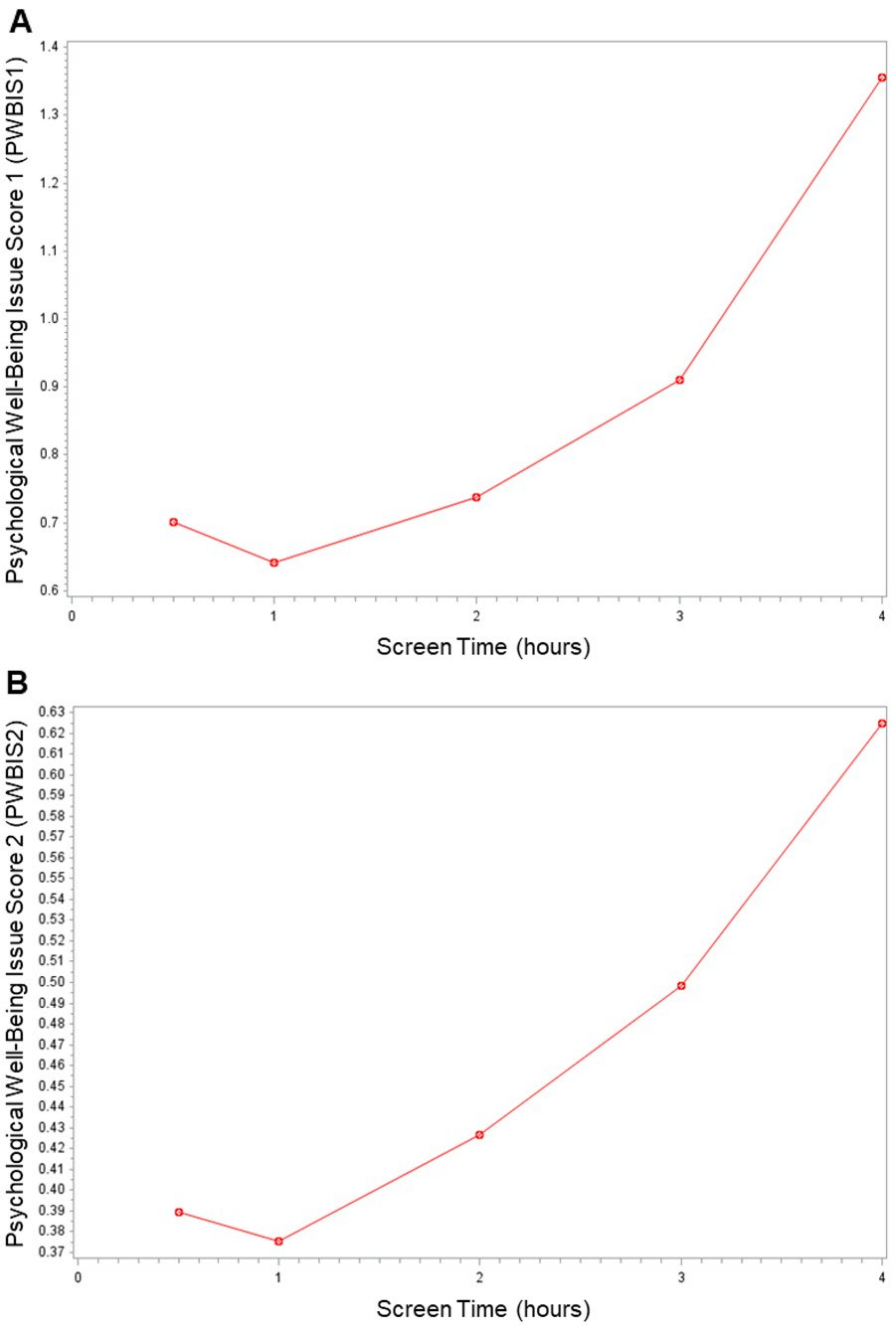
Assessment of recreational screen time by psychological average well-being issue scores for **A** PWBIS1 and **B** PWBIS2

We used the NSCH variable, *SCREENTIME* for reported recreational screen use hours (TV, computer, cellphone, or other electronic devices to watch programs, play games or use social media) on most weekdays. The reported value ranges from 0.5 to 4, where 0.5 represents average screen time of < 1 h, and 4 indicates ≥ 4 h. As previously reported [1], low screen usage (< 1 h) was found to negatively impact PWB, while between 1 and ≥ 4 h, increasing time was associated with worse PWB (Fig. 2). Thus, for the regression analyses, we only included 71,302 observations with at least one hour of screen usage every weekday.

#### Screen overuse assessment

We considered reported recreational screen time during a weekday of ≥ 4 h to be an indicator of screen overuse (variable *Overuse*, yes or no). After accounting for time for school (8 h), sleep (8 h), and commuting, eating and personal time (3–4 h) during a typical school day, there are approximately 4–5 h remaining for the day. We postulated that especially during the pandemic when school was conducted online, ≥ 4 h of recreational screen time indicates that a child is using a screen constantly throughout the day, and there is a reasonable concern of screen overuse. As described above, the maximum survey answer is ≥ 4 h, which is around a similar range to previous studies, including a previous study showing ≥ 3 h per day on social media as a risk factor for mental health among adolescents [10], or a study on screen addiction among children which used ≥ 4 h per weekday as the maximum survey answer [9], or a study on smartphone addiction among university students, which considered ≥ 5 h per weekday as excess use [27].

#### PWBIS development

In a previous study, PWB was described as a broad concept reflective of various factors, including, “emotional stability, positive interpersonal relationships, self-control, and indicators of flourishing as well as diagnoses of mood disorders such as anxiety or depression.” [1] Accordingly, we considered the following NSCH study variables as PWB factors relevant to school-aged children: ability to remain calm and in control when challenged (K7Q85_R), argues too much (K7Q70_R), difficulty making or keeping friends (MAKEFRIEND), works to finish tasks they have started (K7Q84_R), shows interest and curiosity (K6Q71_R), is difficult to care for (K8Q31), has ever been diagnosed by healthcare professionals with depression (K2Q32A) or anxiety (K2Q33A). The original study variables were recoded as dichotomous variables for developing PWB issue scores (PWBIS) for downstream analyses (Additional file 1: Table S1). A child experiencing low PWB is expected to have one or more of the above related psychological symptoms. We constructed two scores to indicate PWB issues (Additional file 1: Table S2) as described below:

***PWBIS1*** is a composite score ranging from 0 to 6, where the higher the value is, the lower the PWB, and calculated by adding six of the PWB symptom dichotomous variables relating to depression and anxiety (*Not Calm*, *Argues Too Much*, *Difficult to Make Friends*, *Does Not Finish Tasks*, *Not Curious*, and *Difficult to Care*).

***PWBIS2*** is a dichotomous score with a value of 1 if any of the eight distinct symptom variables are 1 (*Not Calm*, *Argues Too Much*, *Difficult to Make Friends*, *Does Not Finish Tasks*, *Not Curious*, *Difficult to Care*, *Depression*, or *Anxiety*), or otherwise is 0.

#### Multivariable regression models

Various multivariable Generalized Least Square (GLS) models for PWBIS1 (Model1 and Model2), or logistic regression models for PWBIS2 (Model3 and Model4), were constructed (Additional file 1: Table S3). For each of the four models, either screen time in hours (*Screen Time*) or screen overuse, defined as recreational screen time ≥ 4 h a day (*Overuse*) was included in the model as the main effect to be assessed. Compared to Model1 and Model3, Model2 and Model4 include two additional variables: a dummy variable to represent the pandemic years 2020 and 2021 (*Pandemic years 2020 and 2021*), and an interaction term between the pandemic years dummy variable and screen time or overuse (*Screen Time or Overuse* × *Pandemic years*), to understand how the pandemic years may be an effect modification for the association between recreational screen time and PWBIS. As conventionally conducted in a multi-level model, the centralized and standardized screen time was used to compute the interaction term. Age was categorized as elementary school-age (6–10 years old), middle school-age (11–13 years old), and high school-age (14–17 years old) as the reference group. Categorical variables for sex (female as reference), race (White as reference), and ethnicity (not otherwise Hispanic or Latino as reference) were used.

***Software*** SAS version 9.4 (SAS Institute Inc, NC).

## Results

### Recreational screen time and overuse significantly increased during the pandemic year compared to previous years

Our study included a total of 88,823 school age children (6–17 years old) in the NSCH survey over four years (18,746 in 2018, 18,212 in 2019, 26,253 in 2020, and 25,612 in 2021) (Fig. 1). The overall demographics show that a large number of elementary (6–7 years old), middle (11–13 years old), and high (14–17 years old) school-age children across the US are represented (Table 1). On average, they spent increasing hours of screen recreationally (*i.e.* outside of school work) over the years, with 2.38 h in 2018, 2.41 h in 2019, 2.70 h in 2020, and 2.59 h in 2021 (p < 0.01 compared to each previous year) (Table 2). The proportion of those with screen overuse (hours reported) or overuse (≥ 4 h of recreational screen time a day), also significantly increased over time, with 22.72% in 2018, 24.39% in 2019, 32.80% in 2020, and 29.41% in 2021 (p < 0.01 compared to each previous year), and especially during 2020, the first pandemic year showing a notable 8.41% surge from the previous year (Table 2). The 2020 results also showed a decrease in the proportion reporting ≤ 2 h compared to 2018 and 2019, and an increase in reporting of ≥ 3 h (23.15% in 2018 and 23.79% in 2019, versus 33.86% in 2020), showing an overall changing trend in recreational screen time (p < 0.0001) (Table 3). In addition, the combined amounts from the pre-pandemic years of 2018 and 2019 (23.15% and 23.79%, respectively) are much smaller than the pandemic years of 2020 and 2021 (33.86% and 29.02%).

**Table 1:** Summary of the study population demographics

	N = 88,823
Age group	
Elementary school (age 6–10 years)	31,840(35.84%)
Middle school (age 11–13 years)	21,608 (24.32%)
High school (age 14–17 years)	35,375 (39.82%)
Sex	
Male	45,012 (50.67%)
Female	43,811 (49.32%)
Race	
White	68,899 (77.56%)
Black or African American	6227 (7.01%)
American Indian or Alaska Native	851 (0.96%)
Asian	5002 (5.63%)
Native Hawaiian or Other Pacific Islander	540 (0.60%)
Some other race alone	486 (0.54%)
Two or more races	6818 (7.67%)
Ethnicity	
Hispanic or Latino	10,884 (12.25%)
Otherwise	77,939 (87.75%)
Poverty ratio	292.38 ± 116.82

Categorical demographic variables are shown as n (%) relative to the total population (N = 88,823), and the unweighted Poverty Ratio is shown as mean ± standard deviation (with a range of 50–400)

**Table 2:** Summary of recreational screen time hours and proportion of screen overuse (≥ 4 h)

	Screen time average (hours)				Percentage of screen overuse (≥ 4 h of screen time)			
	2018	2019	2020	2021	2018	2019	2020	2021
All ages (N = 63,211)	2.38	2.41	2.70	2.59	18,746 (22.72%)	18,212 (24.39%)	26,253 (32.80%)	25,612 (29.41%)
*P* value	–	0.0045	< 0.0001	< 0.0001	–	0.0002	< 0.0001	< 0.0001
Elementary school (age 6–10 years) (n = 21,857)	1.98	2.02	2.37	2.21	6478 (11.78%)	6249 (13.55%)	9130 (20.78%)	9983 (17.15%)
*P* value	–	0.0635	< 0.0001	< 0.0001	–	0.0026	< 0.0001	< 0.0001
Middle school (age 11–13 years) (n = 15,634)	2.46	2.47	2.79	2.67	4561 (24.36%)	4564 (24.30%)	6509 (34.59%)	5974 (30.48%)
*P* value	–	0.9190	< 0.0001	< 0.0001	–	0.9455	< 0.0001	< 0.0001
High school (age 14–17 years) (n = 25,720)	2.80	2.86	3.03	3.01	7707 (34.88%)	7399 (38.00%)	10,614 (45.83%)	9655 (43.57%)
*P* value	–	0.0002	< 0.0001	0.0540	–	< 0.0001	< 0.0001	0.0012

Student’s t-test p-values are shown for the difference between the value of the current versus the previous year. Less than 1 h of screen time was recorded as 0.5, and four or more hours was recorded as 4

**Table 3:** Changes in hours of recreational screen time and PWBIS before and during the COVID-19 pandemic

	Total (N = 63,211)	2018 (N = 18,746)	2019 (N = 18,212)	2020 (N = 26,253)	2021 (N = 25,612)
Screen time					
< 1 h	5349 (6.02%)	1315 (7.01%)	1372 (7.53%)	1222 (4.65%)	1440 (5.62%)
1 h	12,172 (13.70%)	3144 (16.77%)	2828 (15.53%)	2803 (10.68%)	3397 (13.26%)
2 h	26,239 (29.54%)	5903 (31.49%)	5726 (31.44%)	7234 (27.55%)	7376 (28.80%)
3 h	20,070 (22.60%)	4045 (21.58%)	3954 (21.71%)	6105 (23.25%)	5966 (23.29%)
4 or more hours	24,993 (28.14%)	4339 (23.15%)	4332 (23.79%)	8889 (33.86%)	7433 (29.02%)
*P* value compared to each previous year	–	–	< 0.0001	< 0.0001	< 0.0001
PWBIS1 (mean)	0.936	0.843	0.839	1.031	1.029
* P* value compared to each previous year	–	–	0.0641	< 0.0001	0.0018
PWBIS2 (%)	48.6%	45.3%	45.8%	51.7%	51.3%
* P* value compared to each previous year	–	–	0.284	< 0.0001	0.385

Chi-square test p-values are shown for the difference between the distribution of the current versus the previous year’s recreational screen time. The mean and Student’s t-test p-values are shown for PWBIS1, and the proportions and Chi-square test p-values are shown for PWBIS2, for differences between the value of the current versus the previous year

### PWB declined significantly during the pandemic

We devised two scores (PWBIS1 and PWBIS2) as indicators of overall mental health, using the NCHS survey variables, allowing an assessment most relevant to children. The composite PWBIS1 considers six NCHS survey questions related to the inability to stay calm, arguing behavior, difficulty in making and maintaining friendship, inability to finish tasks, lack of curiosity, and difficulty for parents to care for. The dichotomous PWBIS2 considers whether any of those six conditions, or additionally, depression or anxiety were reported (Additional file 1: Table S2). While PWBIS1 was comparable between 2018 and 2019 (0.843 and 0.839, respectively, p = 0.0641), it increased significantly in the pandemic year, 2020 (1.031, p < 0.0001 compared to 2019) and 2021 (1.029, p = 0.0018) (Table 3). Similarly, while PWBIS2 score was comparable between 2018 and 2019 (45.3% and 45.8%, respectively, p = 0.284), it increased significantly in 2020 (51.7%, p < 0.0001), and 2021 (51.3%, p = 0.385 compared between 2020 and 2021) (Table 3). These observations demonstrate that the pandemic years indeed had an impact on children’s PWB.

### Screen time and PWB are associated with various demographic factors

In order to further explore potential factors associated with screen time and PWB, we evaluated the distributions of screen time and PWB across sex, race, ethnicity, and poverty ratio. In all age groups (*i.e.* elementary school, middle school, and high school), higher proportions of females were found in the lower < 1 h, 1 h, 2 h screen time categories, while, higher proportions of males were found in the higher 3 h and ≥ 4 h screen time categories (p < 0.0001) (Table 4). For PWBIS1 and PWBIS2 where higher scores indicate worse psychological well-being issue, among all age groups, higher scores were found for males compared with females, while for high school, higher scores were found for females than males.

**Table 4:** Assessment of hours of recreational screen time and PWBIS by various demographic and socioeconomic variables

	Screen time						PWB measures			
	< 1 h	1 h	2 h	3 h	4 or more	*P* value	PWBIS1	*P* value	PWBIS2	*P* value
Elementary school age (6–10 years)										
Female	10.79%	22.46%	34.38%	17.77%	14.60%	< 0.0001	0.81	< 0.0001	44.47%	< 0.0001
Male	9.71%	19.35%	33.39%	20.52%	17.03%		0.95		47.90%	
Black or African American	7.84%	14.65%	32.02%	24.09%	21.39%	< 0.0001	0.81	0.0003	42.28%	< 0.0001
Native American or Alaska Native	5.88%	8.45%	45.54%	24.01%	16.12%	< 0.0001	1.14	< 0.0001	56.53%	< 0.0001
Asian	10.81%	22.16%	28.54%	20.76%	17.73%	< 0.0001	0.85	0.3460	40.30%	< 0.0001
Hawaiian or other Pacific Islander	5.05%	31.77%	34.78%	6.78%	21.62%	< 0.0001	0.77	0.0669	37.90%	< 0.0001
White	11.19%	22.38%	34.06%	18.22%	14.15%	< 0.0001	0.87	0.4136	46.79%	< 0.0001
Some other race alone	7.08%	27.15%	41.30%	15.81%	8.65%	< 0.0001	0.74	0.0339	44.79%	< 0.0001
Two or more races	8.44%	18.07%	35.32%	19.25%	18.91%	< 0.0001	1.02	< 0.0001	50.52%	< 0.0001
Hispanic or Latino related	7.79%	18.18%	34.58%	21.02%	18.42%	< 0.0001	0.91	0.0089	48.26%	< 0.0001
Otherwise	10.99%	21.74%	33.68%	18.56%	15.02%		0.87		45.54%	
Below median poverty ratio	8.95%	19.62%	33.11%	20.54%	17.77%	< 0.0001	0.93	< 0.0001	48.07%	< 0.0001
Above median poverty ratio	12.21%	22.87%	35.06%	17.01%	12.85%		0.8		43.32%	
Middle school age (11–13 years)										
Female	6.09%	13.65%	30.32%	23.34%	26.60%	< 0.0001	0.88	< 0.0001	48.34%	< 0.0001
Male	5.84%	10.64%	28.45%	24.64%	30.43%		1.05		50.16%	
Black or African American	5.75%	10.59%	27.40%	21.64%	34.63%	< 0.0001	0.91	0.0246	46.44%	< 0.0001
Native American or Alaska Native	1.19%	14.89%	23.50%	25.19%	35.22%	< 0.0001	1.12	0.0188	47.15%	< 0.0001
Asian	8.65%	13.11%	29.47%	18.92%	29.85%	< 0.0001	0.77	< 0.0001	43.63%	< 0.0001
Hawaiian or other Pacific Islander	10.47%	10.69%	33.27%	29.31%	16.25%	< 0.0001	1.03	0.4146	52.09%	< 0.0001
White	5.72%	12.61%	30.15%	24.47%	27.06%	< 0.0001	0.98	0.0555	50.12%	< 0.0001
Some other race alone	3.56%	1.18%	40.27%	31.65%	23.35%	< 0.0001	0.96	0.8617	39.71%	< 0.0001
Two or more races	7.20%	12.02%	25.34%	24.03%	31.42%	< 0.0001	1.02	0.0830	51.41%	< 0.0001
Hispanic or latino related	5.34%	10.32%	28.55%	25.47%	30.33%	< 0.0001	0.98	0.4360	50.28%	< 0.0001
Otherwise	6.18%	12.78%	29.68%	23.48%	27.88%		0.96		48.89%	
Below median poverty ratio	6.37%	11.52%	29.03%	24.04%	29.04%	< 0.0001	1.04	< 0.0001	51.42%	< 0.0001
Above median poverty ratio	5.28%	13.21%	29.98%	23.92%	27.62%		0.85		45.60%	
High school age (14–17 years)										
Female	3.69%	8.29%	24.69%	23.16%	40.16%	< 0.0001	0.99	0.2530	52.31%	< 0.0001
Male	3.33%	6.84%	23.66%	25.13%	41.04%		0.98		49.59%	
Black or African American	4.03%	5.29%	21.23%	24.09%	45.36%	< 0.0001	0.97	0.2984	48.20%	< 0.0001
Native American or Alaska Native	2.14%	5.58%	24.61%	23.32%	44.34%	< 0.0001	1.2	< 0.0001	57.65%	< 0.0001
Asian	4.23%	9.94%	21.51%	21.92%	42.40%	< 0.0001	0.81	< 0.0001	46.03%	< 0.0001
Hawaiian or other Pacific Islander	3.02%	9.50%	33.23%	11.66%	42.59%	< 0.0001	0.91	0.1199	49.03%	< 0.0001
White	3.28%	7.84%	24.97%	24.79%	39.12%	< 0.0001	0.98	0.4719	51.24%	< 0.0001
Some Other Race Alone	6.57%	13.48%	17.60%	32.26%	30.09%	< 0.0001	0.76	< 0.0001	47.04%	< 0.0001
Two or more races	3.71%	6.29%	23.50%	22.27%	44.22%	< 0.0001	1.19	< 0.0001	56.82%	< 0.0001
Hispanic or Latino related	3.68%	7.50%	24.06%	22.21%	42.55%	< 0.0001	1.04	< 0.0001	51.55%	< 0.0001
Otherwise	3.46%	7.59%	24.21%	24.80%	39.94%		0.97		50.75%	
Below median poverty ratio	4.16%	7.41%	23.84%	23.20%	41.39%	< 0.0001	1.04	< 0.0001	52.02%	< 0.0001
Above median poverty ratio	2.52%	7.81%	24.68%	25.60%	39.39%		0.9		49.31%	

Proportions and chi-square test p-values are shown for distributions for recreational screen time and PWBIS2. The mean and Student’s t-test p-values are shown for PWBIS1. For the race category comparisons, p-values were calculated for each of the races with all the others. The median poverty ratio among all the samples used in this study was found to be 328, which was used as the cut-off to make comparisons between below and above median poverty ratios

The proportions of the various race categories across screen time were variable among elementary school (5.05% Hawaiian or other Pacific Islander as the lowest and 11.19% White as the highest for the < 1 h category; and 8.65% some other race alone as the lowest and 21.62% Hawaiian or Other Pacific Islander as the highest for the ≥ 4 h category); middle school (1.19% Native American or Alaska Native as the lowest and 10.47% Hawaiian or other Pacific Islander as the highest for the < 1 h category; and 16.25% Hawaiian or other Pacific Islander as the lowest and 35.22% Native American or Alaska Native as the highest for the ≥ 4 h category); and high school (2.14% Native American or Alaska Native as the lowest and 6.57% some other race alone as the highest for the < 1 h category, and 30.09% some other race alone as the lowest and 45.36% Black or African American as the highest for the ≥ 4 h category). A relatively higher proportion of Hispanic or Latino related children were found among the ≥ 4 h screen time category across age groups (18.42% Hispanic or Latino related versus 15.02% otherwise, among elementary school children; 30.33% Hispanic or Latino related versus 27.88% otherwise, among elementary school children; 42.55% Hispanic or Latino related versus 39.94% otherwise, among elementary school children). With regards to PWB measures, for elementary school, Native American or Alaska Native categories were found to have the highest PWBIS1 and PWBIS2 (1.14 and 56.53%). Some Other Race Alone was found as having the lowest PWBIS1 (0.74) and Hawaiian or other Pacific Islander as the lowest PWBIS2 (37.90%). For middle school, Native American or Alaska Native was found to have the highest PWBIS1 (1.12) and Hawaiian or other Pacific Islander as the highest PWBIS2 (52.09%), while Asian was found having the lowest PWBIS1 (0.77) and some other race alone as the lowest PWBIS2 (39.71%). For high school, Native American or Alaska Native was found with both the highest PWBIS1 and PWBIS2 (1.2 and 57.65%). Some Other Race Alone was found as having the lowest PWBIS1 (0.76) and Asian aas the lowest PWBIS2 (46.03%).

As there are various definitions for poverty level, in this study, we evaluated the median poverty ratio of the overall study as the cut off and compared the screen time above and below the median poverty ratio. For all age groups (*i.e.* elementary school, middle school, and high school), higher proportions were found in the below median poverty ratio groups for ≥ 4 h screen time categories (p < 0.0001), while the results for the lower < 1 h, 1 h, 2 h and 3 h screen time categories were variable. For all age groups, below median poverty ratio was found to have higher PWBIS1 and PWBIS2, compared to above median, as expected.

### Recreational screen time and overuse are associated with declining PWB, and the pandemic years significantly strengthened the association

As previously suggested [1], low screen usage (< 1 h) resulted in reduced PWB, while increasing time was associated with worse PWB when evaluating screen usage between 1 and ≥ 4 h, (Fig. 2). Therefore, we performed regression analyses, with only 71,302 observations with at least one hour of screen usage every weekday. We constructed various multivariable GLS models to evaluate the magnitude of the association between recreational screen time (in hours) or overuse (≥ 4 h a day) and PWBI1, adjusting for age, race, ethnicity, and poverty ratio, first without considering the 2020 and 2021 pandemic years (Model1), and separately, including the pandemic years as a covariate in the model or as an interaction term (Model2) to assess its impact as an effect measure modifier (Table 5). Both screen time and overuse were positively associated with PWBI1, and the models including the 2020 and 2021 pandemic years as a covariate also showed significant association or effect modification (p < 0.001 in all models).

**Table 5:** GLS models 1 and 2 regression results for PWBIS and screen time or addition

PWBIS1 outcome	Effect of screen time				Effect of overuse (*i.e.* Screen time ≥ 4 h)			
	Model 1		Model 2		Model 1		Model 2	
Covariates	β (95% CI)	*P* value	β (95% CI)	*P* value	β (95% CI)	*P* value	β (95% CI)	*P* value
*Intercept*	0.340 (0.290, 0.40)	< 0.001	0.330 (0.270, 0.390)	< 0.001	1.070 (1.030, 1.120)	< 0.001	1.050 (1.00, 1.100)	< 0.001
*Screen time or overuse*	0.320 (0.310, 0.330)	< 0.001	0.320 (0.310, 0.330)	< 0.001	0.570 (0.550, 0.590)	< 0.001	0.570 (0.540, 0.590)	< 0.001
*Pandemic years (2020–21)*	NA	NA	0.070 (0.040, 0.090)	< 0.001	NA	NA	0.080 (0.050, 0.110)	< 0.001
*Interaction term (Model2): Screen time or overuse* × *Pandemic years (2020–21)*	NA	NA	0.000 (− 0.030, 0.030)	0.878	NA	NA	− 0.01 (− 0.06, 0.030)	0.626
*Age*	− 3.58E-3 (− 6.59E-3, − 5.77E-4)	0.019	− 3.27E−3 (− 6.28E−3, − 2.60E−4)	0.033	− 1.47E−3 (− 4.46E−3, 1.52E−3)	0.336	− 1.16E−3 (− 4.15E−3, 1.83E-3)	0.447
*Sex-male (ref: female)*	0.060 (0.040, 0.080)	< 0.001	0.060 (0.040, 0.080)	< 0.001	0.070 (0.050, 0.090)	< 0.001	0.070 (0.050, 0.090)	< 0.001
*Black or African American (ref. White)*	− 0.200 (− 0.230, − 0.170)	< 0.001	− 0.200 (− 0.230, − 0.170)	< 0.001	− 0.200 (− 0.230, − 0.170)	< 0.001	− 0.200 (− 0.230, − 0.170)	< 0.001
*Native American or Alaska Native* *(ref. White)*	0.200 (0.130, 0.280)	< 0.001	0.200 (0.130, 0.270)	< 0.001	0.200 (0.130, 0.270)	< 0.001	0.200 (0.130, 0.270)	< 0.001
*Asian (ref. White)*	− 0.190 (− 0.240, − 0.150)	< 0.001	− 0.190 (− 0.240, − 0.150)	< 0.001	− 0.190 (− 0.240, − 0.150)	< 0.001	− 0.190 (− 0.240, − 0.150)	< 0.001
*Hawaiian or other Pacific Islander (ref. White)*	− 0.040 (− 0.110, 0.040)	0.317	− 0.050 (− 0.120, 0.030)	0.219	− 0.070 (− 0.150, 0.00)	0.067	− 0.080 (− 0.160, 0.00)	0.037
*Some other race alone (ref. white)*	− 0.130 (− 0.210, − 0.050)	0.002	− 0.110 (− 0.190, − 0.030)	0.008	− 0.120 (− 0.200, − 0.040)	0.004	− 0.100 (− 0.180, − 0.020)	0.019
*Two or more races (ref. white)*	0.090 (0.060, 0.130)	< 0.001	0.09 (0.060, 0.130)	< 0.001	0.090 (0.050, 0.130)	< 0.001	0.090 (0.050, 0.120)	< 0.001
*Hispanic or Latino related (ref. otherwise)*	− 0.070 (− 0.100, − 0.050)	< 0.001	− 0.070 (− 0.100, − 0.050)	< 0.001	− 0.070 (− 0.100, − 0.050)	< 0.001	− 0.070 (− 0.100, − 0.050)	< 0.001
*Poverty ratio*	− 9.04E−4 (− 9.89E-4, − 8.19E-4)	< 0.001	− 9.07E−4 (− 9.92E−4, − 8.23E−4)	< 0.001	− 9.07E−4 (− 9.92E−4, − 8.22E-4)	< 0.001	− 9.10E− 4 (− 9.95E−4, − 8.25E − 4)	< 0.001
Adjusted R squared	4.78%		4.82%		4.76%		4.81%	

These analyses were performed using 71,302 observations of subjects with at least one hour of screen usage every weekday on average

Using a similar approach, we also constructed multivariable logistic regression models for the PWBI2 outcome, adjusting for age group, sex, race, ethnicity, and poverty ratio. Each hour increase in screen time was associated with 1.498 times increased odds (p < 0.001), and screen overuse, was associated with 2.0 times (p < 0.001) the odds of PWBIS2 outcome, without considering the pandemic years (Model3) (Table 6). Including the pandemic years and their interaction term, screen hours was associated with an OR of 1.489, and screen overuse with an OR of 1.978, for the PWBIS2 outcome (Model4). In these models, the pandemic years were associated with an OR of 1.094 for the model with screen time, or 1.102 for the model with overuse. The interaction terms also showed a positive significant association (p < 0.001), suggesting the impact of the pandemic on strengthening the effect of screen time and overuse on PWB issues.

**Table 6:** Odds ratio (OR) estimates of multivariable logistic regression models for PWBIS and screen time or overuse

PWB1S2 outcome	Effect of screen time				Effect of overuse (*i.e.* Screen time ≥ 4 h)			
	Model 3		Model 4		Model 3		Model 4	
Covariates	OR (95% CI)	*P* value	OR (95% CI)	*P* value	OR (95% CI)	*P* value	OR (95% CI)	*P* value
*Screen time or overuse*	1.498 (1.497, 1.500)	< 0.001	1.489 (1.488, 1.490)	< 0.001	2.000 (1.997, 2.003)	< 0.001	1.978 (1.974, 1.981)	< 0.001
*Pandemic years (2020–21)*	*NA*	*NA*	1.094 (1.092, 1.096)	< 0.001	*NA*	*NA*	1.102 (1.100, 1.105)	< 0.001
*Interaction term (Model4): Screen time or overuse* × *pandemic years*	*NA*	*NA*	1.012 (1.010, 1.014)	< 0.001	*NA*	*NA*	1.021 (1.018, 1.024)	< 0.001
*Elementary school (ref. high school)*	1.016 (1.015, 1.018)	< 0.001	1.013 (1.011, 1.015)	< 0.001	0.992 (0.990, 0.993)	< 0.001	0.989 (0.987, 0.990)	< 0.001
*Middle School (ref. High School)*	1.044 (1.042, 1.046)	< 0.001	1.042 (1.040, 1.044)	< 0.001	1.040 (1.038, 1.042)	< 0.001	1.038 (1.036, 1.039)	< 0.001
*Sex-male (ref. female)*	1.016 (1.015, 1.018)	< 0.001	1.016 (1.014, 1.017)	0.030	1.008 (1.006, 1.009)	0.002	1.007 (1.006, 1.008)	< 0.001
*Black or African American (ref. White)*	0.688 (0.687, 0.69)	< 0.001	0.688 (0.687, 0.69)	< 0.001	0.693 (0.692, 0.695)	< 0.001	0.694 (0.692, 0.695)	< 0.001
*Native American or Alaska Native (ref. White)*	1.147 (1.142, 1.153)	< 0.001	1.141 (1.135, 1.147)	< 0.001	1.145 (1.139, 1.151)	< 0.001	1.138 (1.132, 1.144)	< 0.001
*Asian (ref. White)*	0.712 (0.709, 0.714)	< 0.001	0.711 (0.709, 0.714)	< 0.001	0.714 (0.711, 0.716)	< 0.001	0.714 (0.711, 0.716)	< 0.001
*Hawaiian or other Pacific Islander (ref. White)*	0.926 (0.921, 0.931)	< 0.001	0.914 (0.91, 0.919)	0.146	0.888 (0.884, 0.893)	< 0.001	0.877 (0.872, 0.882)	< 0.001
*Some Other Race Alone(ref. White)*	0.900 (0.895, 0.905)	< 0.001	0.923 (0.918, 0.929)	0.013	0.909 (0.904, 0.915)	0.062	0.935 (0.930, 0.940)	< 0.001
*Two or More Races (ref. White)*	1.162 (1.159, 1.165)	< 0.001	1.161 (1.158, 1.164)	< 0.001	1.160 (1.158, 1.163)	< 0.001	1.159 (1.156, 1.162)	< 0.001
*Hispanic or latino related (ref. otherwise)*	0.911 (0.909, 0.913)	< 0.001	0.909 (0.907, 0.911)	< 0.001	0.915 (0.913, 0.917)	< 0.001	0.913 (0.911, 0.915)	< 0.001
*Poverty ratio*	0.999 (0.999, 0.999)	< 0.001	0.999 (0.999, 0.999)	< 0.001	0.999 (0.999, 0.999)	< 0.001	0.999 (0.999, 0.999)	< 0.001

These analyses were performed using 71,302 observations of subjects with at least one hour of screen usage every weekday on average

Collectively, these various results show that higher screen time and overuse are significantly related to various measures of PWBIS over 2018–2021, and that the pandemic years of 2020 and 2021 contributed to children’s low PWB. Furthermore, the interaction terms between screen time or overuse and the years 2020 and 2021 highlight the impact of the pandemic on strengthening the effect of screen time or overuse on PWB issues.

## Discussion

The NCHS dataset used in this study included a large sample of school-age children across the entire US, providing results that are more widely applicable compared to previous studies. Previous studies explored the impact of the pandemic years on the overall (*i.e.* instructional and recreational combined) screen use [11, 12] or their associations with mental health only among a subset of children from a narrower age range who previously reported severe COVID-related family and economic hardship [13] or worry [14]. Therefore, our objective was to perform a more comprehensive study comparing the years before versus during the pandemic, to evaluate the effect, specifically of recreational screen overuse, among all school-age children (6–17 years old) and all demographics and economic status generally across the US, rather than specific groups of children. These differences underscore the wide applicability, importance, and novelty of our findings.

Among our study’s large population, we quantified the significant surge in recreational screen time and overuse and decline in PWB during the 2020 and 2021 pandemic years, compared to prior years of 2018 and 2019. We developed various PWBIS that are relevant to children, and assessed their association with recreational screen time and overuse in various regression models, as well as constructed additional models to demonstrate the impact of the pandemic on PWB independently of screen time or overuse, and also found evidence that it strengthens the association between screen use and PWB. We also identified differences in both screen time and PWB scores comparing various demographic variables of sex, race, ethnicity, and socioeconomic status to investigate subgroup differences. Additional investigations of possible long-term impact and studies in other nations could also be informative.

Innovations and decreasing costs have made online education, entertainment, and virtual social connections increasingly more accessible and equitable [28], and during the pandemic, access to education and social connections through screens was vital to children’s mental health [29]. A previous study conducted several years before the pandemic suggested that while excessive internet use is well-established to be associated with depression, too little usage hours could also be associated with depression [30]. Our study using data of two years leading up to, and during the pandemic, also found that screen use of less than one hour resulted in worse PWB compared to one hour, while increasing hours beyond one hour showed worsened PWB. Despite the benefits from reasonable use, excessive screen use is a major concern especially among children, as reduced recreational activities that involve observations and explorations of the environment can negatively impact cognitive development [31].Excessive screen time is also associated with adverse physiological effects, including obesity [32, 33], sleep disruption, [2, 33–36] and cardiovascular, vision and skeletal problems [2], biochemical imbalances [2], depression and inattention problems, depression, anxiety, and poor self-esteem [7, 9, 10, 35–38]; and on cognitive functioning, including language development [39], executive and academic performance [9, 33], attention span [40], and hindered social interactions from a sedentary lifestyle [41]. Taken together, these results suggest that optimal screen time exposure that allows sufficient remote social interactions and entertainment, while limiting excess use is key, and further studies to identify the optimal length of time that is advantageous for PWB is informative.

This study has several limitations pertaining to the use of the NSCH dataset used and analysis methods. The 2020 data collection occurred between June 2020 and January 2021 [42], during which most children were undergoing only online instruction; however, it is not possible to determine the exact timing when each study participant responded to the survey questions, and variability in the phase of the pandemic is expected. Moreover, the survey answer choices on recreational screen hours are designed such that ≥ 4 h is the maximum recorded response, and anything above is classified as the same response. Additionally, self-reported average hours on a weekday was the response recorded, although in reality, usage hours are expected to be variable between days. Furthermore, some of the survey questions gathered the parents’ responses, rather than the children’s. Another limitation is in the lack of an established definition for problem use versus addiction [22, 43], and in our study, we considered reported use of ≥ 4 h of recreational (*i.e.* not school-related) screen time during school days as excessive use, as it suggests that after accounting for school, sleep, commute and feeding time, a child is spending the rest of the day on a screen. From the current dataset’s survey questions [44], it is not possible to ascertain whether these children have psychological dependency on screen devices and other indicators of addiction, and future studies with survey questions or clinical evaluations directly assessing screen addiction, with follow-up to evaluate persistence of the behavior would be beneficial. Finally, compared to surveys administered before the pandemic when children were physically in school, self-reported recreational screen time may be more likely to be overestimated during the pandemic when online instruction was used.

Despite the limitations above, our study finding strong associations between screen time or overuse and PWB, and the significant impact of the pandemic on strengthening these associations, using a large survey dataset collected widely across the US is important. Future studies to evaluate whether these associations persist post-pandemic, and investigations in other regions are also informative. Prevention of screen overuse by incorporating other activities, such as sports, music, arts, and social hours [45] off screens is expected to improve children’s PWB. Practical implications from our study may include recommending parents to discourage excessive screen time that dominate adolescents’ after school schedules. Previous studies reporting that parental mediation can be protective of [46], and on the other hand, negative parenting is associated with [9] dependency and decreased PWB supports this notion. For educators, while it is necessary to integrate technology into learning and assignments, it may also be important to include other modes of learning tools to help adolescents have periodic screen-free time. Finally, for policymakers, while it is important to promote technology and make it available to all children, it is equally important to encourage additional research on screen overuse to better understand its impact on PWB, and elucidate additional protective factors.

### Supplementary Information

**Additional file 1: Table S1.** Description of NSCH survey questions and answer choices and variables used in this study. **Table S2.** Description of the construction of the well-being scores, PWBIS1 and PWBIS2. **Table S3.** Description of the GLS and logistic regression models constructed.

## Data Availability

Datasets were downloaded from the National Survey of Children’s Health (NSCH) website, https://www.census.gov/programs-surveys/nsch/data/datasets.html.

## Abbreviations

NSCH: National Survey of Children’s Health
PWB: Psychological well-being
PWBIS: Psychological well-being issue score
COVID-19: Coronavirus disease of 2019
