# Supplementary material for: Change in screen time and overuse, and their association with psychological well-being among US-wide school-age children during the COVID-19 pandemic: analysis of the National Survey of Children’s Health (NSCH) years 2018–21
Source: Child Adolesc Psychiatry Ment Health. 2024 Jan 13;18:9. doi: 10.1186/s13034-023-00688-7 (PMC10787397; doi:10.1186/s13034-023-00688-7)
Supplement: Supplementary file 1 — Additional file 1: Table S1. Description of NSCH survey questions and answer choices and variables used in this study. Table S2. Description of the construction of the well-being scores, PWBIS1 and PWBIS2. Table S3. Description of the GLS and logistic regression models constructed. [file 13034_2023_688_MOESM1_ESM.pdf]

# Change in Screen Time and Overuse, and their Association with Psychological Well-being Among US-wide School-Age Children During the COVID-19 Pandemic: Analysis of the National Survey of Children's Health (NSCH) Years 2018-21

Helena T. Wu[a,b], Jiandong Li, PhD[c], and Amy Tsurumi, PhD[b,d,e*]

**Affiliations:**

[a]University of Chicago, Chicago, IL, USA

[b]Department of Surgery, Massachusetts General Hospital, Boston, Massachusetts, USA

[c]Central University of Finance and Economics, Beijing, China

[d]Harvard Medical School, Boston, Massachusetts, USA

[e]Shriners Hospitals for Children-Boston, Boston, Massachusetts, USA

*Corresponding Author: Amy Tsurumi

(atsurumi@mgh.harvard.edu; 50 Blossom St., Their 340, Boston, MA 02114, USA)

| **NSCH survey variable (variable name according to the public use data file) and detailed description of the question and answer choices on the original survey** | **Recoded variable used in this study** |
|---|---|
| **Recreational screen time (SCREENTIME).**<br>"On most week days, about how much time did this child spend in front of a TV, computer, cellphone or other electronic device watching programs, playing games, accessing the internet or using social media, not including school work?"<br>Answer choices: 1 = Less than 1 hour, 2 = 1 hour, 3 = 2 hours, 4 = 3 hours or 5 = 4 or more hours. | ***Screen Time***<br>0.5 = SCREENTIME of 1 (*i.e.* Less than 1 hour),<br>1 = SCREENTIME of 2 (*i.e.* 1 hour),<br>2 = SCREENTIME of 3 (*i.e.* 2 hours),<br>3 = SCREENTIME of 4 (*i.e.* 3 hours),<br>4 = SCREENTIME of 5 (*i.e.* 4 or more hours). |
| **Ability to remain calm and in control when challenged (K7Q85_R).**<br>"How often: Does this child stay calm and in control when faced with a challenge?"<br>Answer choices: 1 = Always, 2 = Usually, 3 = Sometimes, 4 = Never. | ***Not Calm***<br>1 = K7Q85_R value of 3 or 4, indicating the child does not stay calm or in control,<br>0 = K7Q85_R value of 1 or 2, indicating the child does stay calm or in control. |
| **Children who argue too much (K7Q70_R).**<br>"How often: Does this child argue too much?"<br>Answer choices: 1 = Always, 2 = Usually, 3 = Sometimes, 4 = Never. | ***Argues Too Much***<br>1 = K7Q70_R1 value of 1 or 2, indicating the child argues too much,<br>0 = K7Q70_R1 value of 3 or 4, indicating the child does not argue too much. |
| **Difficulty making or keeping friends (MAKEFRIEND).**<br>"Compared to other children his or her age, how much difficulty does this child have making or keeping friends?"<br>Answer choices: 3 = A lot of difficulty, 2 = A little difficulty, 1 = No difficulty. | ***Difficult to Make Friends***<br>1 = MAKEFRIEND of 2 or 3, indicating the child has difficulty making or keeping friends,<br>0 = MAKEFRIEND of 1, indicating the child does not have difficulty making or keeping friends. |
| **Children who work to finish the tasks they start (K7Q84_R).**<br>"How often: Does this child work to finish tasks they start?"<br>Answer choices: 1 = Always, 2 = Usually, 3 = Sometimes, 4 = Never. | ***Does Not Finish Tasks***<br>1 = K7Q84_R value of 3 or 4, indicating the child does not finish tasks,<br>0 = K7Q84_R value of 1 or 2, indicating the child does finish tasks. |
| **Children who show interest and curiosity in learning new things (K6Q71_R).**<br>"How often: Does this child show interest and curiosity in learning new things?"<br>Answers choices: 1 = Always, 2 = Usually, 3 = Sometimes, 4 = Never. | ***Not Curious***<br>1 = K6Q71_R value of 3 or 4, indicating the child does not show interest or curiosity,<br>0 = K6Q71_R value of 1 or 2, indicating the child does show interest and curiosity. |
| **Parent felt a child is difficulty to care for (K8Q31).**<br>"During the past month, how often have you felt: that this child is much harder to care for than most children his or her age?"<br>Answers choices: 1 = Never, 2 = Rarely, 3 = Sometimes, 4 = Usually, 5 = Always. | ***Difficult to Care***<br>1 = K8Q31 value of 3, 4 or 5, indicating the child is difficult to care for,<br>0 = K8Q31 value of 1 or 2, indicating the child is not difficult to care for. |
| **Been diagnosed with depression (K2Q32A)**<br>"Has a doctor or other health care provider EVER told you that this child has depression?" Answers choices: 1 = Yes, 2 = No. | ***Depression***<br>1 = K2Q32A value of 1, indicating a doctor or other health care provider has diagnosed that the child has depression,<br>0 = K2Q32A value of 2, indicating the child has not been diagnosed with depression. |
| **Been diagnosed with anxiety (K2Q33A)**<br>"Has a doctor or other health care provider EVER told you that this child has anxiety?" Answers choices:1 = Yes, 2 = No. | ***Anxiety***<br>1 = K2Q33A value of 1, indicating the child ever had anxiety,<br>0 = K2Q33A value of 2, indicating the child has never had anxiety. |

**Table S1: Description of NSCH survey questions and answer choices and variables used in this study**

| PWIS | Values | Definition |
|---|---|---|
| PWBIS1 | 0 - 6 | Sum of six symptom dichotomous variables excluding depression and anxiety (*Not Calm, Argues Too Much*, *Difficult to Make Friends*, *Does Not Finish Tasks, Not Curious*, and *Difficult to Care*) |
| PWBIS2 | 1 or 0 | 1 = has at least one of the eight symptoms (*Not Calm*, *Argues Too Much*, *Difficult to Make Friends*, *Does Not Finish Tasks, Not Curious*, *Difficult to Care*, *Depression*, or *Anxiety*), or<br>0=none of the symptoms |

**Table S2: Description of the construction of the well-being scores, PWBIS1 and PWBIS2.**

| GLS models for the PWBIS1 outcome | |
|---|---|
| **Model1** | $PWBIS1_{Model1} = \alpha + \beta_1$ Screen Time/Overuse + $\beta_2$ Age + $\beta_3$ Sex-male + $\beta_4$ Race-Black<br>+ $\beta_5$ Race-American Indian or Alaska native + $\beta_6$ Race-Asian<br>+ $\beta_7$ Race-Hawaiian or Pacific Islander + $\beta_8$ Race-Other + $\beta_9$ Race-Two or More Races<br>+ $\beta_{10}$ Ethnicity-Hispanic or Latino + $\beta_{11}$ Poverty Ratio + $\mathcal{E}$ |
| **Model2** | $PWBI1S_{Model2} = \alpha + \beta_1$ Screen Time/ Overuse + $\beta_2$ Pandemic 2020 + $\beta_3$ Screen Time/ Overuse*Pandemic 2020<br>+ $\beta_4$ Age + $\beta_5$ Sex-male + $\beta_6$ Race-Black + $\beta_7$ Race-American Indian or Alaska native<br>+ $\beta_8$ Race-Asian + $\beta_9$ Race-Hawaiian or Pacific Islander + $\beta_{10}$ Race-Other<br>+ $\beta_{11}$ Race-Two or More Races + $\beta_{12}$ Ethnicity-Hispanic or Latino + $\beta_{13}$ Poverty Ratio + $\mathcal{E}$ |
| **Logistic regression models for the PWBIS2 outcome** | |
| **Model3** | $PWBIS2_{Model3} = \alpha + \beta_1$ Screen Time/ Overuse + $\beta_2$ Elementary School + $\beta_3$ Junior School + $\beta_4$ Sex-male<br>+ $\beta_5$ Race-Black + $\beta_6$ Race-American Indian or Alaska Native + $\beta_7$ Race-Asian<br>+ $\beta_8$ Race-Hawaiian or Pacific Islander + $\beta_9$ Race-Other + $\beta_{10}$ Race-Two or More Races<br>+ $\beta_{11}$ Ethnicity-Hispanic or Latino + $\beta_{12}$ Poverty Ratio + $\mathcal{E}$ |
| **Model4** | $PWBIS2_{Model4} = \alpha + \beta_1$ Screen Time/ Overuse + $\beta_2$ Pandemic 2020 + $\beta_3$ Screen Time/ Overuse*Pandemic 2020<br>+ $\beta_4$ Elementary School + $\beta_5$ Junior School + $\beta_6$ Sex-male + $\beta_7$ Race-Black<br>+ $\beta_8$ Race-American Indian or Alaska Native + $\beta_9$ Race-Asian<br>+ $\beta_{10}$ Race-Hawaiian or Pacific Islander + $\beta_{11}$ Race-Other + $\beta_{12}$ Race-Two or More Races<br>+ $\beta_{13}$ Ethnicity-Hispanic or Latino + $\beta_{14}$ Poverty Ratio + $\mathcal{E}$ |

**Table S3: Description of the GLS and logistic regression models constructed.**
